# Free energy surface of initial cap formation in carbon nanotube growth

**DOI:** 10.1039/d1na00377a

**Published:** 2021-09-10

**Authors:** Satoru Fukuhara, Yasushi Shibuta

**Affiliations:** Department of Materials Engineering, The University of Tokyo 7-3-1 Hongo, Bunkyo-ku Tokyo 113-8656 Japan shibuta@material.t.u-tokyo.ac.jp +81-3-5841-7118

## Abstract

Initial cap formation is an important process of carbon nanotube (CNT) growth where a hexagonal carbon network is lifted off from the catalyst surface. In this study, free energy surface (FES) of initial cap formation in the CNT growth is investigated by metadynamics simulation. A two-dimensional collective variable (CV) space is newly developed to examine the complicated formation process of the cap structure, which consists of the formation of a hexagonal carbon network and lift-off of the network from the catalyst surface. States before and after the lift-off of the carbon network are clearly distinguished in the two-dimensional FES. Therefore, free energy difference before and after the lift-off can be directly derived from the two-dimensional FES. It was revealed that the cap structure is stable at a high temperature due to the entropy effect, while the carbon network covering the catalyst surface is energetically stable. The new insight in this study is achieved owing to metadynamics simulation in conjunction with a newly developed two-dimensional CV space since it is impossible to explore FES for such complicated processes in the framework of conventional molecular dynamics simulation.

## Abstract 2

Free energy surface of the formation of a carbon nanotube cap is investigated by metadynamics simulation with a two-dimensional collective variable space.

## Graphical abstract

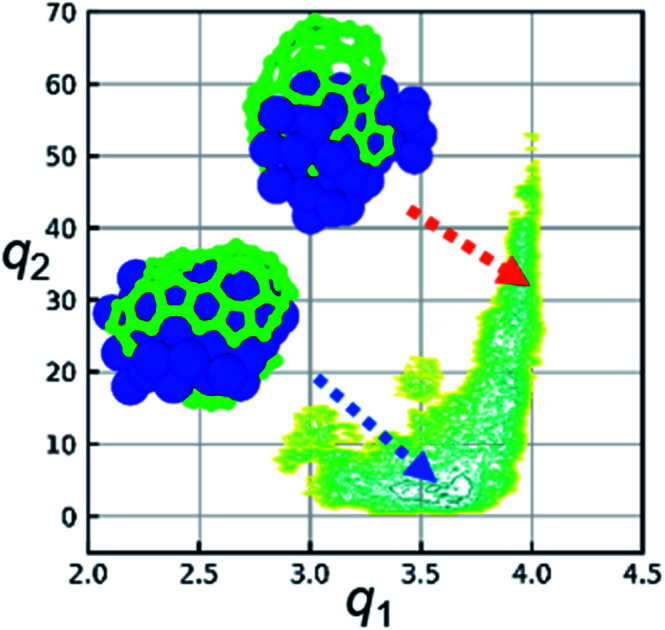

## 1. Introduction

Carbon nanotubes (CNTs)^1^ can potentially be used in a variety of applications.^2^ It is essential to control the diameter and chirality of CNTs during the synthesis for better application. The catalytic chemical vapor deposition (CCVD) method^2^ is widely used to synthesize CNTs, in which CNTs grow from dispersed nanoparticles.^3^ Diameter and chirality of CNTs are strongly correlated with the properties of catalytic nanoparticles at the initial stage of the synthesis process. Therefore, it is important to understand the role of catalytic nanoparticles at the initial stage of CNT growth. Molecular dynamics (MD) simulations^4–9^ have contributed to the understanding of the metal-catalyzed growth mechanism of CNTs from an atomistic viewpoint. In conjunction with *in situ* transmission electron microscopy (TEM) observations,^10,11^ the initial growth process of CNTs by CCVD is considered as follows:^12^ aggregation of carbon atoms in/on the catalytic nanoparticle, formation of the hexagonal carbon network on the surface of catalytic nanoparticles and the lift-off of the carbon network to form a cap structure.

In general, an MD simulation represents one specific trajectory starting from an initial configuration and does not cover all the ensemble space. It is theoretically possible to cover all the free energy surface of target phenomena if the MD simulation is performed for a sufficiently long time. However, it is not realistic since the time scale of MD simulation is limited to the nanosecond order at most. Therefore, most of the MD simulations of the CNT growth were limited as an enormous part of possible trajectories are cut out^3,4^ and it is not clear that the discussion based on one-time MD simulation covers the universal feature of CNT growth. To overcome this problem, metadynamics^13^ is proposed in which the efficiency of the sampling of target phenomena is improved by setting collective variables (CVs) and applying a bias to the CVs. Target phenomena can be efficiently sampled in conjunction with appropriate CVs. Up to now, numerous CVs have been developed for various phenomena such as bond dissociation reaction,^14^ ligands docking on receptors,^15^ atomic diffusion^16^ and solid–liquid phase transition.^17^ For example, a bond length between atoms is used as a CV for describing bond dissociation and docking reactions. In these simulations, a bond for the target reaction is pre-defined and its length is simply used as CV. However, it is not straightforward to set appropriate CVs for complex and multi-step phenomena. For example, the initial formation process of CNTs consists of the formation of a carbon dimer, junction and hexagonal network, and subsequent lift-off of the network to form a cap structure. We previously developed a CV based on the coordination number of carbon atoms to discuss a part of the initial formation process of CNTs and we sampled the potential energy surface of carbon segregation from a nickel nanoparticle by metadynamics simulation.^18^ Although the states of isolated atoms, dimer, junction, and triple junction was properly distinguished, it was not successful to cover the subsequent formation process of a hexagonal carbon network and lift-off of the network to form a cap structure using one unified CV. To this end, a two-dimensional CV space representing the formation of a hexagonal carbon network and lift-off of the network was newly developed in this study. Using the newly developed CVs, the free energy surface (FES) of the cap formation process of CNTs is investigated by metadynamics simulation.

## 2. Methodology

### 2.1. Free energy calculation with metadynamics

First, a method to calculate the FES by MD simulation is described. The FES for CV **q** is obtained by sampling the appearance probability *P*(**q**) of **q** as1
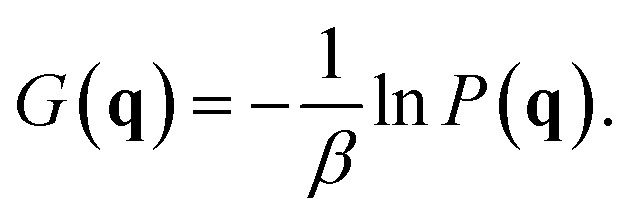
Here, *β* is 1/*k*_B_*T*, where *k*_B_ is the Boltzmann constant and *T* is the temperature. In an ergodic system, *P*(**q**) can be obtained by simply recording a histogram *N* as function of **q** as eqn (2) with sufficiently long MD calculation.^19^2
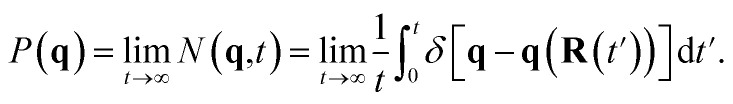


The total free energy of state A, *G*_A_, is calculated as:3
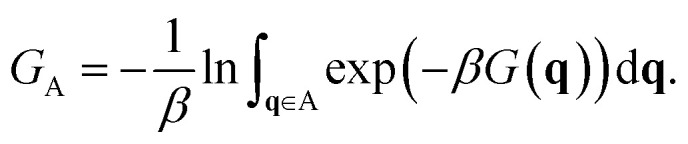


However, it is not straightforward to achieve sufficient sampling within the time scale of normal MD simulation.

In this study, metadynamics was employed to improve the efficiency of sampling. In metadynamics, a bias potential in the form of Gaussian functions with width *σ* and height *W* defined as:4
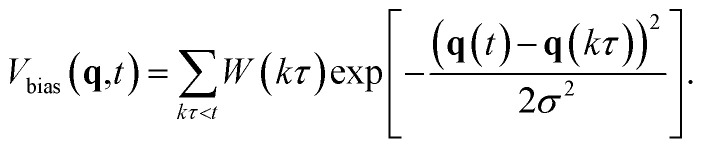
is added to the CV at every *kτ*, where *k* is an integer and *τ* is the interval. The height *W* is modified based on the well-tempered metadynamics^20^ scheme as5
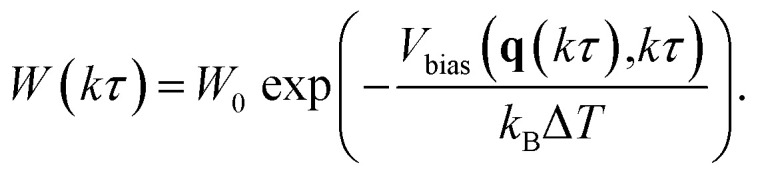
Here, *W*_0_ is the initial height and Δ*T* is calculated from a bias factor *γ* as6
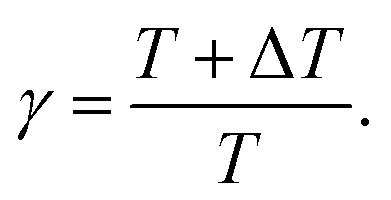


*P*(**q**) can be obtained by creating a histogram weighed by

7 exp(*βV*_bias_(**q**) − *βc*(*t*))

during the calculation.^21^ Here, *c*(*t*) is defined as8
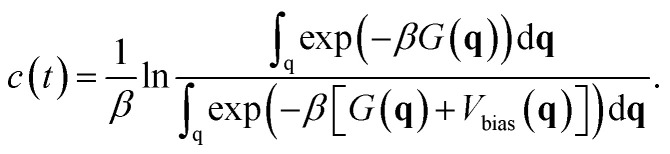


### 2.2. Two-dimensional collective variable space for the formation of a carbon nanotube cap

**Fig. 1:**
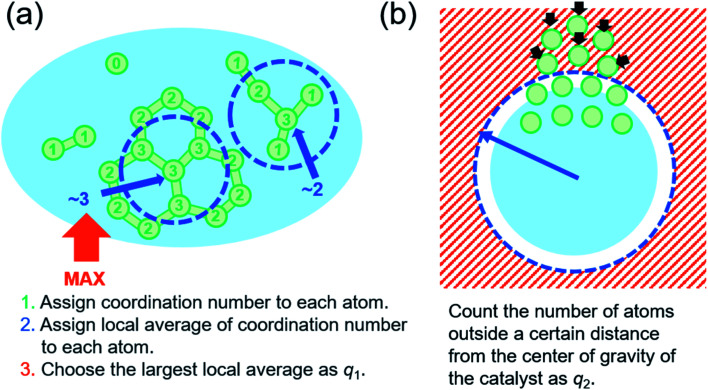
(a) Schematic of the definition of a collective variable, *q*_1_. Green circles represent carbon atoms with the numeric value indicating the coordination number. Blue circles with the numeric value represent the local average of the coordination number. (b) Schematic image of the definition of a collective variable *q*_2_. Green circles represent carbon atoms and light blue circle represents the catalyst. Carbon atoms outside a certain distance from the center of gravity of the catalyst are counted as the lifted-off atoms.

As described in the introduction, it is most important to define appropriate CVs for metadynamics simulation. In this study, a two-dimensional CV space is newly developed to represent the formation process of the cap structure of CNT. This consists of the formation of a hexagonal carbon network on the surface of a catalytic nanoparticle and the lift-off of the network from the surface. It is essential to distinguish two states before and after the lift-off of the carbon network from the catalyst surface. The hexagonal carbon network covers the catalyst surface in the former (hereinafter called covering-state) and the carbon network is lifted off from the catalyst surface forming a cap structure in the latter (hereinafter called cap-state). The first CV *q*_1_ was defined to describe the network formation. The definition consists of the following three steps (see Fig. 1(a)). (i) Coordination number *c*_*i*_ of carbon atom *i*, for other carbon atoms *j* is defined using switching function *s* as9
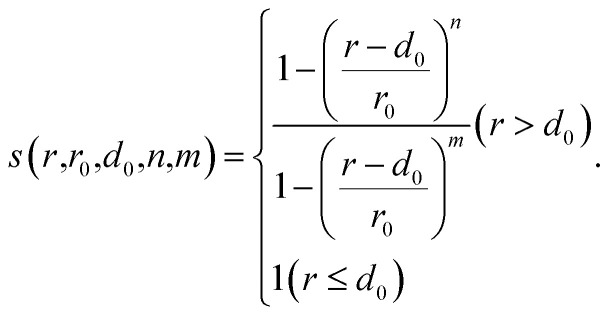
10
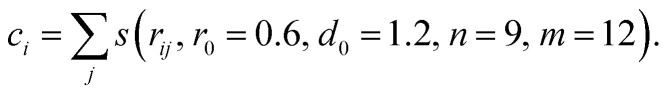
Here, *r*_*ij*_ is the distance between atoms *i* and *j*. Cutoff parameters *d*_0_ and *r*_0_ represent offset distance and width at half maximum, respectively. Exponents *n* and *m* represent the degree of decay of switching function at the cutoff distance. Parameters in eqn (10) are employed from our previous work.^15^ Coordination numbers of 0, 1, 2 and 3 represent isolated atom, atom in dimer, atom in chain and atom in the center of triple junction, respectively. (ii) The local average of the coordination number is calculated since the coordination number is not sufficient to distinguish the atom in the center of a triple junction from one in the connected hexagonal network. The local average *l*_*i*_ of the coordination number around atom *i* is calculated using the weighting factor *w*_*ij*_ as11
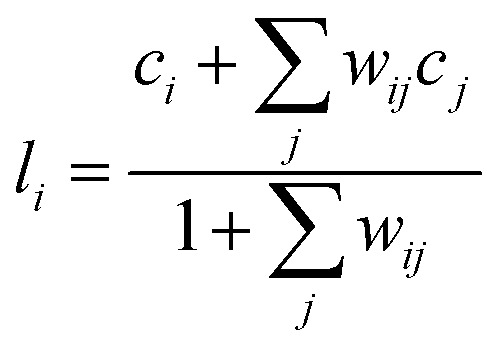

12 *w*_*ij*_ = *s*(*r*_*ij*_, *r*_0_ = 4.0, *d*_0_ = 0, *n* = 6, *m* = 12).

The local average of the coordination number for the atom in the complete hexagonal network is assigned as 3, whereas that of the imperfect hexagonal network is assigned as less than 3, as shown in Fig. 1(a). Therefore, the local average of the coordination number can distinguish these structures correctly. (iii) The largest value of local averages of coordination number assigned to each atom is selected as the first CV *q*_1_. Since the CV must be a continuous value to add bias in metadynamics, eqn (13) is employed for the simulation.13
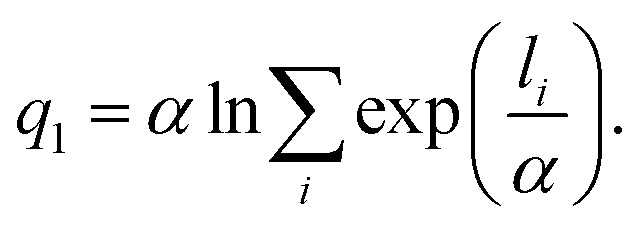
*α* = 0.01 is used in this study.

The second CV *q*_2_ is defined to represent the lift-off state of the carbon network structure from the nanoparticles. The number of atoms located above a certain distance from the center of gravity of the catalyst counted using switching function is employed as14
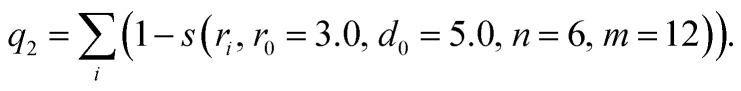
Here, *r*_*i*_ is the distance between atom *i* and the center of the gravity of the catalyst. The second CV *q*_2_ was used only to distinguish states whether the hexagonal carbon network is lifted-off or not. Note that *q*_2_ is not used to accelerate the reaction itself.

### 2.3. Considered systems and conditions

Metadynamics simulation of the cap formation process of CNTs is performed using above defined two CVs. The simulation methodology basically follows our previous work^18^ except for the definition of CVs and the calculation system. A Ni_55_C_110_ nanoparticle placed at the center of a 35.24 × 35.24 × 35.24 Å^3^ cubic cell is employed as the initial configuration. The nanoparticle is prepared by annealing Wulff-shaped Ni_55_ and randomly placed 110 carbon atoms at 2000 K for 100 ps first and then at 1500 K for 200 ps. Ni was chosen as an element of the catalytic metals since it is one of the most popular catalysts used in the CNT synthesis and is a target of numerous previous computational works.^3,4,8,9,18^ The number of Ni atoms is set as 55 to make the particle diameter around 1 nm, which is consistent with the diameter of a small single-walled CNT. It was confirmed from our previous simulation that the number of carbon atoms must be at least larger than that of Ni atom for spontaneous precipitation.^22^ Therefore, the number of carbon atoms was set as 110, which is large enough to form the initial cap structure. Repulsive Gaussians of height *W*_0_ = 0.043 eV and width *σ* = 0.05 were employed as biasing parameters in eqn (5). Bias potential is added at every 200 steps. A bias factor of 200 was used for the well-tempered metadynamics scheme. A repulsive wall potential (spring constant of 830 eV) was set to prevent carbon atoms from getting too close to each other and it also acts on atoms with the largest value of coordination number *c*_*i*_ when it exceeds 4.2. A ReaxFF reactive force field^23^ was employed to describe the interaction between nickel and carbon atoms, which has already been successfully applied to simulations of CNT nucleation and growth.^8,9^ The NVT (the number of atoms, cell size and temperature constant) ensemble was sampled using a Nosé–Hoover thermostat.^24,25^ Simulations were carried out for seven different temperatures, *viz.* 1000, 1250, 1500, 1750, 2000, 2250 and 2500 K. 3.0 × 10^7^ simulation steps were carried out for all cases with a time step of 0.25 fs. All calculations were performed using a Large-scale Atomic/Molecular Massively Parallel Simulator (LAMMPS)^26^ with PLUMED plugin.^27^

## 3. Results and discussion

### 3.1. Validation of metadynamics simulation

**Fig. 2:**
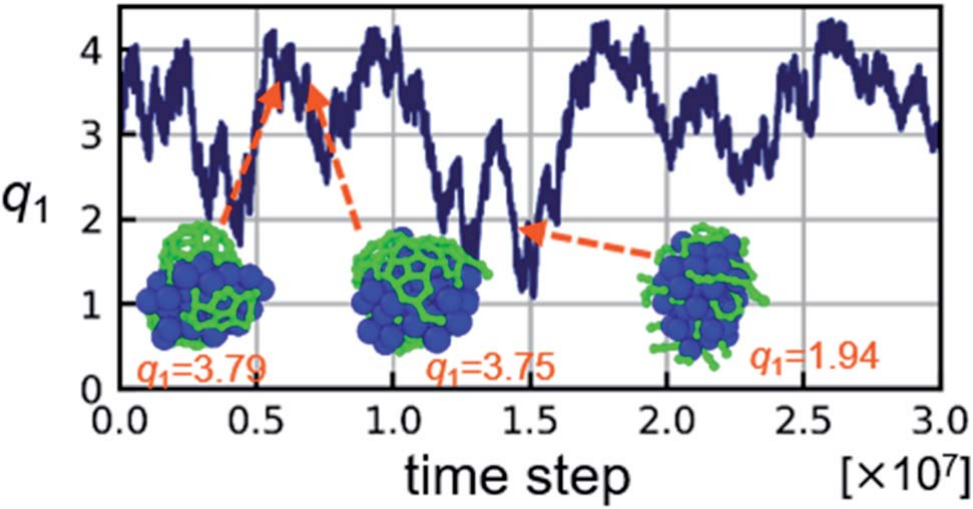
Time evolution of the value of collective variable *q*_1_ during the metadynamics simulation at 2000 K. Insets represent snapshots of the atomic configuration of the system at the time indicated by the arrow.

Fig. 2 shows the time evolution of CV, *q*_1_ for the metadynamics simulation at 2000 K. The value of *q*_1_ oscillates between one and four. Insets represent snapshots of the atomic configuration of the system at the time indicated by the arrow. Cap- and covering-states appear at the time of high *q*_1_ values, whereas the state without the carbon network appears at the time of low *q*_1_. Therefore, the oscillation of *q*_1_ with respect to time indicates the repetition of the formation and disappearance of the hexagonal carbon network during the metadynamics simulation. In the normal MD simulation, reactions usually proceed in one direction and the reverse reaction is rarely observed. That is, the hexagonal carbon network does not disappear once it forms^4,8^ in the normal MD simulation. Therefore, this oscillation verifies that the metadynamics using *q*_1_ successfully accelerated the sampling of possible states including the hexagonal carbon network in the metadynamics simulation. Note that the value of *q*_1_ can exceed three since carbon atoms further than the bond length can have a finite contribution to the coordination number due to the definition of *q*_1_ as having a continuous value.

### 3.2. Free energy surface of cap formation

**Fig. 3:**
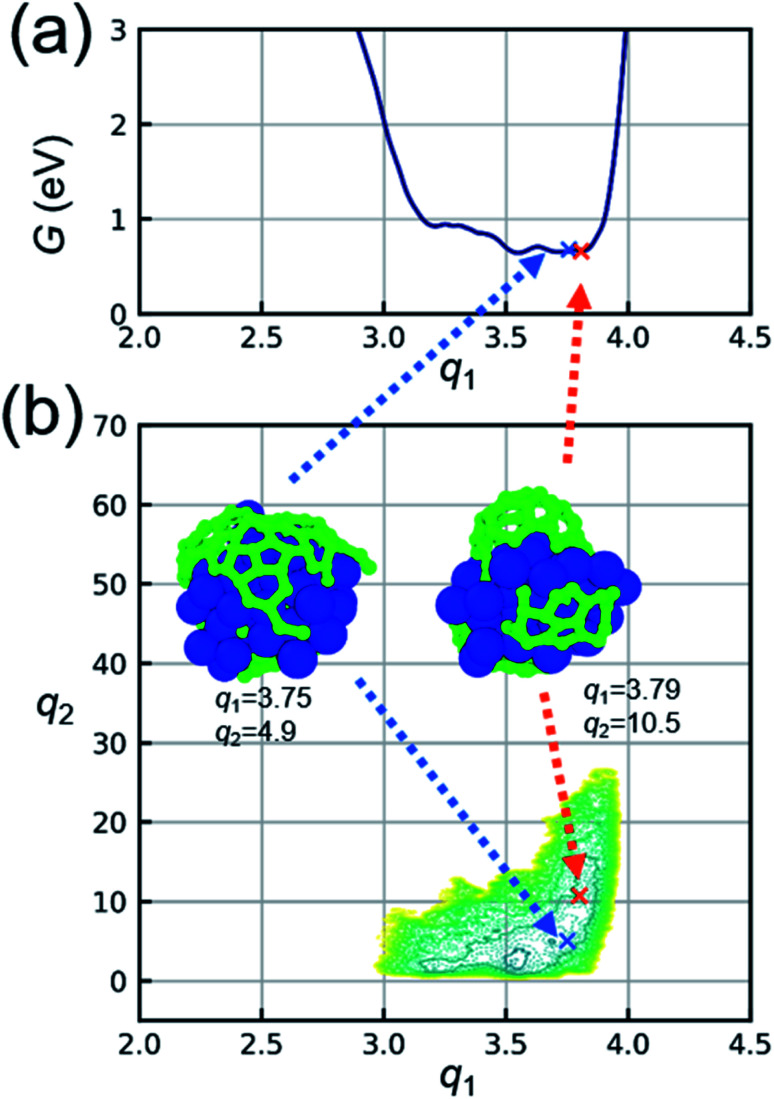
(a) Free energy surface (FES) along the collective variable (CV) *q*_1_ and (b) two-dimensional FES along CVs *q*_1_ and *q*_2_ for the metadynamics simulation at 2000 K. Insets show representative snapshots of covering-state (blue arrow) and cap-state (orange arrow) at the value of CVs indicated by the arrow.

Fig. 3(a) shows the FES along *q*_1_ obtained from the metadynamics simulation at 2000 K. The FES indicates that the states between *q*_1_ = 3.6 and *q*_1_ = 3.8 are the most stable. The insets in Fig. 3 show representative structures for the covering- and cap-states. The values of *q*_1_ for both structures are close to 3.8 and therefore it is not able to distinguish these two structures by *q*_1_ only. Therefore, FES is expanded to a two-dimensional space using *q*_2_. Fig. 3(b) shows the two-dimensional FES along *q*_1_ and *q*_2_ obtained from the metadynamics simulation at 2000 K. Note that the bias potential is added to *q*_1_ only during the metadynamics simulation and *q*_2_ is just recorded during the simulation. Therefore, the simulation itself is the same even if two CVs are recorded. The states successfully separated into different spaces in the two-dimensional FES. That is, covering- and cap-states around *q*_1_ = 3.8 spread out horizontally in the two-dimensional FES.

**Fig. 4:**
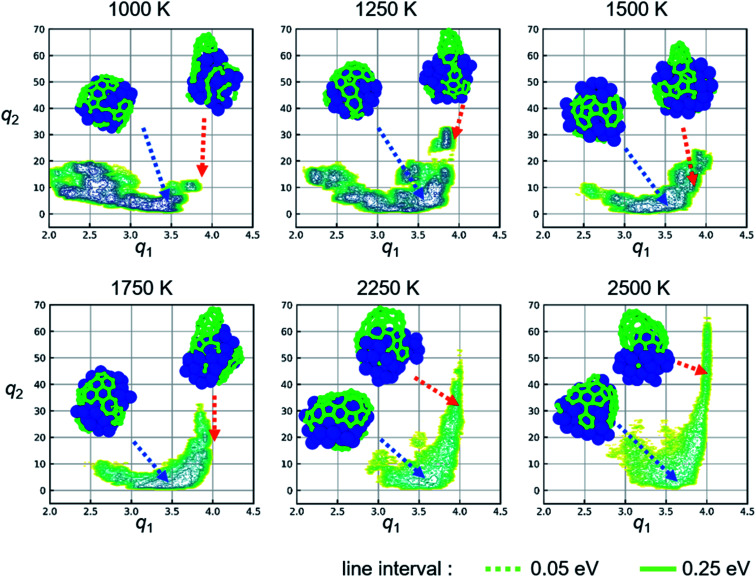
Two-dimensional free energy surfaces (FESs) obtained from metadynamics simulations at various temperature conditions. Insets show representative snapshots of covering-state (blue arrow) and cap-state (orange arrow) at the value of CVs indicated by the arrow.

Fig. 4 shows two-dimensional FES obtained from metadynamics simulations at other temperature conditions. The insets in Fig. 4 show representative structures for the covering-state and cap-state. Both covering- and cap-states are observed in the separated region of two-dimensional FES at all temperature conditions. As the temperature increases, the region of stable state in FES distributes upward at around *q*_1_ = 4. The value of *q*_2_ does not exceed 20 at 1000 K, whereas the values of *q*_2_ exceed 40 at 2250 K and 60 at 2500 K. Moreover, the area of cap-state in FES becomes large with an increase in temperature. This means that the large cap structure is more stable at high temperatures, which agrees with the direct observation of snapshots from the simulation. For example, the cap structure in the snapshot at 1000 K (*q*_2_ = 15.4) has a diameter of approximately 4 Å, while that at 2500 K (*q*_2_ = 43.7) corresponds to a diameter of approximately 10 Å. Regarding the growth mode, perpendicular growth (*i.e.*, the line contact for the catalyst-tube contact) appears at the Ni catalyst with a high carbon concentration in our simulation. It agrees with the previous report^28^ that a high carbon concentration inside the metal particle favors perpendicular growth mode, whereas low carbon fractions in the catalyst lead to tangential growth mode (*i.e.*, the surface contact). Note that the diameter of the cap structure is also affected by the diameter of the catalyst, which is however out of the scope of this study.

### 3.3. Free energy difference between covering-state and cap-state

**Fig. 5:**
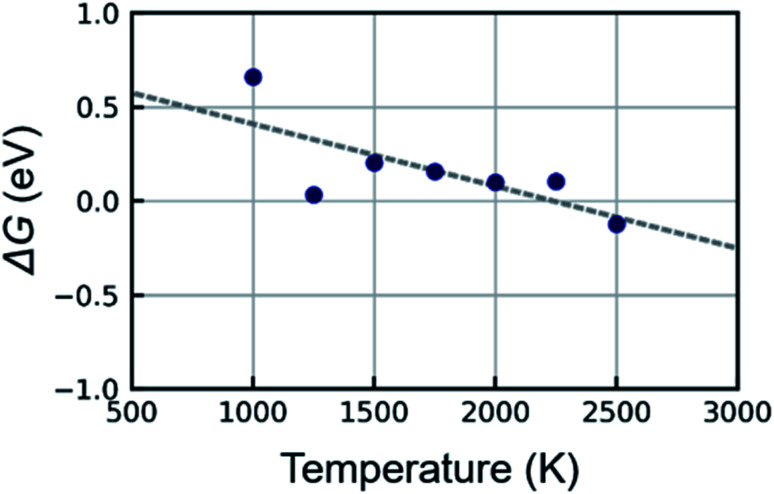
Free energy difference between covering- and cap-states, Δ*G* = *G*_cap_ − *G*_covering_ as a function of temperature. The dashed line represents the linear fitting.

Next, the free energy difference between the covering- and cap-states is investigated. The free energy of each state can be derived from eqn (3) once the region of each state is defined in FES. Here, the region of *q*_1_ ≥ 3.4 and *q*_2_ < 7.0 in FES is regarded as the covering-state and that of *q*_1_ ≥ 3.7 and *q*_2_ ≥ 7.0 is regarded as the cap-state. The free energy difference Δ*G* is calculated by the difference between the free energy of the covering- and cap-states as Δ*G* = *G*_cap_ − *G*_covering_. Fig. 5 shows the free energy difference between the covering- and cap-states as a function of temperature. A positive value of Δ*G* indicates the free energy of the covering-state is lower (*i.e.*, more stable) than that of cap-state and *vice versa*. As shown in the figure, the covering-state is more stable than the cap-state at a low temperature and Δ*G* becomes small with an increase in temperature. The cap-state becomes more stable than the covering-state at 2500 K. The line fitted to the plots corresponds to the equation Δ*G* = Δ*H* − *T*Δ*S* according to the definition in thermodynamics, where Δ*H* and Δ*S* represent enthalpy difference and entropy difference between covering- and cap-states, respectively. From the vertical intercept and the slope of the fitted line, Δ*H* and Δ*S* are estimated to be 0.74 eV and 0.33 × 10^−3^ eV/*T*, respectively. This suggests that the covering-state is enthalpically (*i.e.*, energetically) stable due to the positive value of Δ*H*. However, the cap-state can be stable at high temperatures since entropy effect becomes dominant *via* the term −*T*Δ*S*. This result makes sense since the hexagonal carbon network covering the catalyst surface has more catalyst-carbon bonds than the network lifted-off from the surface. The catalyst-carbon bonds make the covering-state more stable energetically. In contrast, the cap structure is entropically stable since there is no limit to the number of atoms consisting of the cap structure, whereas there is a geometric constraint in the arrangement of the carbon network covering the nanoparticle surface. In fact, the region of the stable cap state is spread out along the *q*_2_ axis in FES at 2500 K, which indicates that cap structures consisting of numerous atoms are sampled in the metadynamics simulation. This result provides a reasonable answer to the long-standing issue that the lift-off of the cap structure is hard to occur at low temperatures^5^ from a thermodynamic viewpoint.

## 4. Conclusion

Initial cap formation process of the CNT growth is investigated by metadynamics simulation on a two-dimensional CV space. Newly developed two-dimensional CV successfully distinguished states of the hexagonal carbon network covering on the catalyst surface and that lifted-off from the catalyst surface in the FES. The free energy difference between the covering- and cap-states with respect to temperature suggests that the cap-state is stable at high temperatures due to the entropy effects, while the covering-state is energetically stable. These findings suggest appropriate directions for efficient CNT synthesis and the formation of the CNT cap is suitable at a higher temperature although the lower temperature is in general preferred as it prevents the nanoparticles from aggregating. Moreover, our result provides a reasonable answer to the long-standing issue that the lift-off of the cap structure is hard to occur at low temperatures. In the practical synthesis process, additional carbons are supplied throughout the reaction process although the number of carbon atoms is fixed in this study. It is expected that the degrees of freedom of carbon atoms for the cap structure will increase as the number of carbon atoms increases. The cap structure is expected to be more stable from an entropy viewpoint with increasing degree of freedom even at lower temperatures. The effect of the number of carbon atoms as well as the effect of the size of catalytic nanoparticles will be examined in the next study.

## Data availability

The data that support the findings of this study are available from the corresponding author upon reasonable request.

## Conflicts of interest

There are no conflicts to declare.

## Supplementary Material
